# Supplementary material for: Nickel-Containing Ceria-Zirconia Doped with Ti and Nb. Effect of Support Composition and Preparation Method on Catalytic Activity in Methane Dry Reforming
Source: Nanomaterials (Basel). 2020 Jun 30;10(7):1281. doi: 10.3390/nano10071281 (PMC7407892; doi:10.3390/nano10071281)
Supplement: Supplementary file 1 [file nanomaterials-10-01281-s001.pdf]

Turnover frequency (TOF) is defined as the maximum number of chemical conversions of substrate molecules N_{CH_4} per unit time over a single catalytic site:

$$TOF = \frac{N_{CH_4}}{N_{Ni} \cdot t}, \tag{1}$$

where N_{Ni} is number in surface nickel cites

The following transformations could be done:

$$TOF = \frac{N_{CH_4} \cdot N_A}{N_{Ni_{surf}} \cdot t \cdot N_A} = \frac{\nu_{CH_4}}{\nu_{Ni} \cdot t} = \frac{\nu_{CH_4} \cdot V}{\nu_{Ni} \cdot t \cdot V} = \frac{C_{CH_4}}{C_{Ni} \cdot t}, \tag{2}$$

where N_A is Avohadro's number 6.02 × 10^{23},

ν_{CH_4} is amount of substance of methane, mol,

ν_{Ni} is amount of substance of surface nickel, mol,

V is volume, L,

C_{CH_4} is methane concentration, mol L^{-1}

C_{Ni} is surface nickel concentration, mol L^{-1}

Since

$$C_{Ni_{surf}} = N_{Ni} \cdot \rho_{cat}, \tag{3}$$

and

$$W_0 = \frac{C_{CH_4}}{t}, \tag{4}$$

then

$$TOF = \frac{C_{CH_4}}{N_{Ni} \cdot \rho_{cat} \cdot t}, \tag{5}$$

$$TOF = \frac{W_0}{N_{Ni} \cdot \rho_{cat}}, \tag{6}$$

where N_{Ni} is surface nickel concentration, mol g^{-1} of catalyst

ρ_{cat} is density of catalyst, g L^{-1}

W_{CH_4} is reaction rate, mol L^{-1} s^{-1}
